# Safe drilling zones for anteriorly, central, and posteriorly angulated syndesmotic stabilization devices

**DOI:** 10.1007/s00167-022-07291-x

**Published:** 2022-12-22

**Authors:** S. F. Baumbach, A. Synek, F. T. Spindler, L. Bauer, W. Böcker, H. Polzer

**Affiliations:** 1 Department of Orthopaedics and Trauma Surgery, Musculoskeletal University Center Munich (MUM), University Hospital, LMU Munich, Ziemssenstraße 5, 80336 Munich, Germany; 2 Institute of Lightweight Design and Structural Biomechanics, TU Wien, Vienna, Austria

**Keywords:** Suture button system, Syndesmosis, Syndesmotic injury, Stabilization

## Abstract

**Purpose:**

The purposes of the study were to (1) analyze the shape of the distal fibula at the location of syndesmotic stabilization and to (2) define safe zones at the distal-lateral fibula for three different drilling tunnel orientations: anteriorly-, posteriorly angulated and center-center.

**Methods:**

Postoperative, bilateral CT images of adult patients that underwent syndesmotic stabilization (suture-button system) for an acute, unilateral ankle injury were analyzed. Manual axial CT reconstructions of the uninjured side were generated. First, the axial shape of the distal fibula was classified. The aspect ratio between the anterio-lateral and the posterior-lateral surfaces of the fibula was calculated to assess symmetry. Second, the same axial planes were used to define the safe zones. Each drilling-tunnel orientation (anterior, central, posterior) comprised a fixed medial tibial anchor point and a safe zone on the lateral fibula. For each of the three orientations, the most anteriorly and posteriorly drilling tunnel location was simulated. Next to a cumulative visual analysis, a quantitative analysis of the most anterior and posterior point on the anterio- and posterior-lateral surfaces was calculated.

**Results:**

A total of 96 CT datasets were analyzed. (1) 81% of fibulae revealed a triangular convex-, 10% an irregular-, and 8% a quadrilateral shape. The lateral surface ratio was 1.0 ± 0.2 (range: 0.7–1.5), not differing between the fibula types (n.s.). (2) The safe corridor on the lateral surface of the fibula for an anteriorly angulated drilling tunnel was − 8% to − 41%, for a posteriorly angulated drilling tunnel was 0% to 46%, and for a center-center alignment − 7 ± 11% (range: − 28 to 18%).

**Conclusion:**

The meta-diaphyseal region of the distal fibula revealed a homogeneous crosssectional shape. The lateral apex of the fibula can serve as a landmark defining safe zones to place the drilling tunnels correctly. Applying these safe zones in clinical practice could help to avoid the misplacement of the syndesmotic fixation device.

**Level of evidence:**

Level III, retrospective radiographic study.

**Supplementary Information:**

The online version contains supplementary material available at 10.1007/s00167-022-07291-x.

## Introduction

The syndesmosis resembles a dynamic three-point fixation of the fibula to the tibia and comprises the anterior inferior tibiofibular ligament (AiTFL), the interosseous membrane (IOM), and the posterior inferior tibiofibular ligament (PiTFL) [1]. Unstable syndesmotic injuries require surgical treatment [2]. Still there is an ongoing discussion on how to diagnose, grade, and treat unstable syndesmotic injuries. Even for the treatment, uniform recommendations on which implants to use and how to place these implants are missing [3, 4].

In general, it is recommended to place a syndesmotic screw parallel to the plafond, 2–3 cm proximal to the ankle joint, along the central axis in the axial plane [5–8]. Suture-button systems are most often placed similarly. However, an increasing number of authors have recommended to angulate the suture-button system according to the syndesmotic structures injured. In case of a two-ligament injury (AiTFL and IOM), the suture-button system should be angulated anteriorly. The same applies in trimalleolar ankle fractures if the posterior malleolus has been addressed by open reduction and internal fixation. If all three syndesmotic ligaments are ruptured (AiTFL, IOM, and PiTFL) numerous authors recommend the use of two angulated suture-button systems [9, 10].

Due to the small diameter of the fibula, there is a risk of placing the drilling tunnel eccentrically within the fibula. This can result in a considerable weakening of the anterior or posterior fibular cortex and subsequent peri-osteosynthetic fractures. Therefore, it is of great interest to identify the optimal entry point (safe zones) at the lateral aspect of the distal fibula for anteriorly-, posteriorly angulated and center-center placed suture-button systems.

To define a uniform entry point at the lateral aspect of the fibula, the shape of the distal fibulae must be comparable or at least present with comparable landmarks which could guide the orientation. Previous studies have so far only assessed the cross-sectional shape of the diaphyseal aspect of the fibula [11–14] but not at the level of the distal articulation between tibia and fibula.

Therefore, the aims of the current study were two-fold: (1) Analyzing the shape of the distal fibula at the location of syndesmotic stabilization; (2) Defining safe zones at the distal-lateral fibula for an anteriorly-, posteriorly angulated and center-center placed suture-button system. Clearly defined drilling-tunnel safe zones can help the surgeon to reduce the risk of eccentric drilling, i.e. weakening the cortex of the fibula.

## Materials and methods

The retrospective radiographic and computational study was approved by the local university hospital ethic committee (IRB ID number: 22-0314 KB).

### Patient selection

The patient selection was based on a previously published cohort [15]. In brief, all adult patients that underwent syndesmotic stabilization with a suture-button system for an acute, unilateral ankle injury with postoperative bilateral CT imaging between 2010 and 2020 were identified at a single academic, level 1 trauma center. The type of injury, i.e. an ankle fracture or isolated syndesmotic instability, were of no matter. The suture-button system used was the TightRope^®^ (Fa. Arthrex, Naples, FL, USA). Excluded were all patients with signs of a previous injury or osteoarthritic changes on the contralateral ankle joint on CT imaging. CT images must have had a resolution of at least 0.70 mm slice thickness to allow for further analysis.

Out of 732 patients, 304 were treated with a suture-button system, and 147 patients had postoperative, bilateral CT imaging. In 98 patients the postoperative CT had a sufficient resolution. Two patients were excluded due to posttraumatic changes on the contralateral side resulting in 96 eligible patients. The patients’ mean age was 37 ± 14 years, 34% were female, and the left side was affected in 54%. Injury types consisted of fractures with syndesmotic injuries in 56% and isolated syndesmotic injuries (including bony avulsion of the AITFL) in 44% of patients. The distal tibiofibular joint was stabilized by a single/double suture-button system in 73%/19% of patients. 8% were treated using a suture-button system and a syndesmotic screw.

### Shape of the distal fibula

**Fig. 1:**
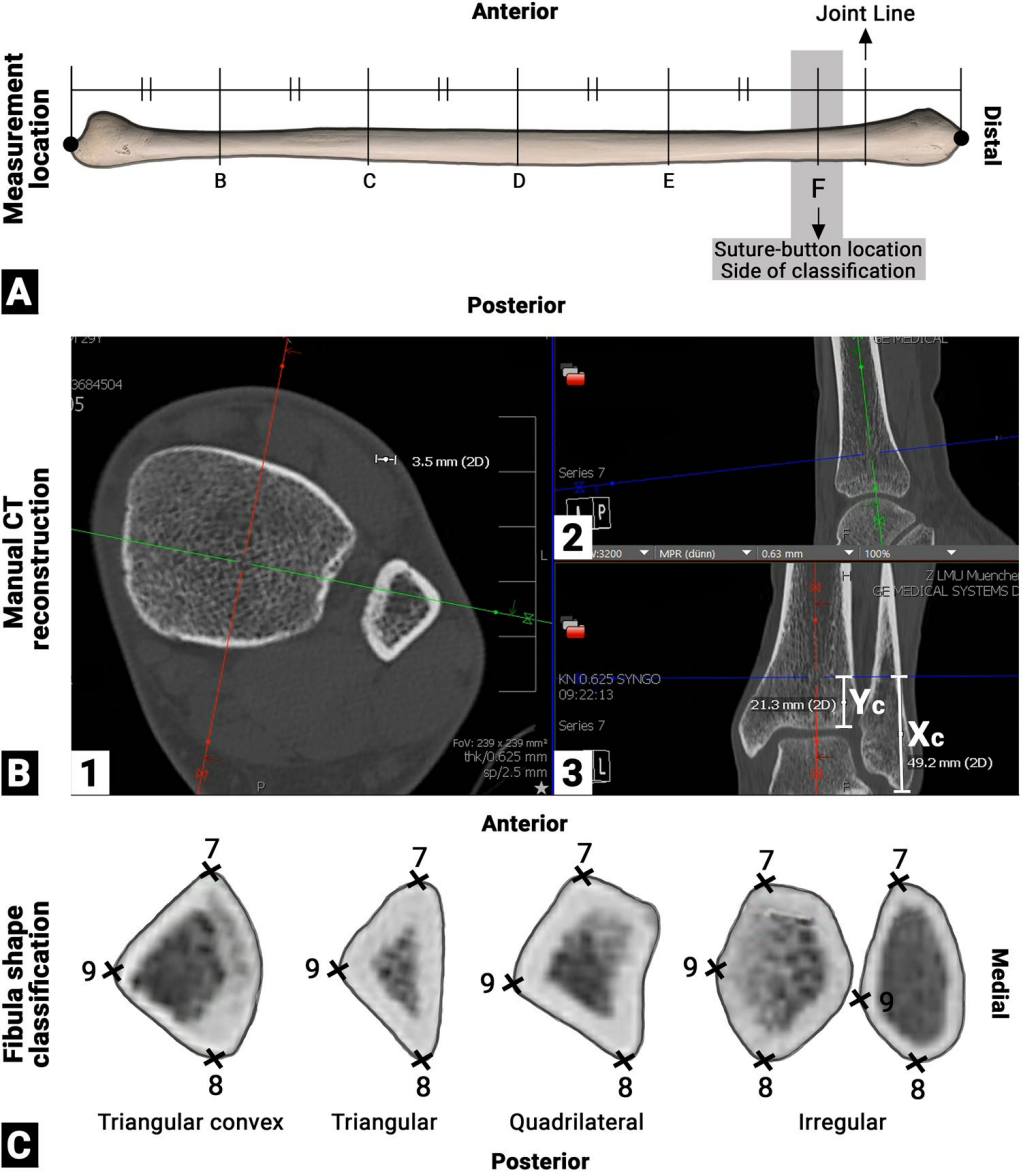
**A** Measurement location of the shape of the fibula **B** manual reconstruction of the CT datasets at the height of the suture button **C** different shapes of the fibula. F: measurement location for the shape of the distal fibula and the suture-button location of the contralateral, uninjured side; joint line: distal pilon tibiale; triangular convex: triangular shape with a convex hypotenuse; triangular: truly triangular shaped fibular; quadrilateral: more rectangular shaped with four edges; irregular: all other shapes, including round or pentagonal shapes; 7: anterior cortical apex; 8: posterior cortical apex; 9: lateral cortical apex; Yc: height of the measurement location from the distal lateral pilon tibial to the proximal end of the articulation between the distal tibia and fibula (suture-button location); Xc: height of the measurement location from the distal tip of the fibula to the proximal end of the articulation between the distal tibia and fibula (suture-button location)

A more detailed description of the methodology applied is given in Supplement 1. The shape of the distal fibula was evaluated on the postoperative CT images of the contralateral, uninjured side. First, the CT data sets were manually reconstructed (Fig. 1B) and an axial slice parallel to the tibial plafond was generated at the preferred location for a suture-button system (Fig. 1B1). Then, the height of the axial slices was measured from the most lateral aspect of the tibia plafond (Fig. 1B3: Yc) and from the tip of the distal fibula (Fig. 1B3: Xc). All reconstructed images were saved as.jpg files.

Next, the shape of the distal fibula was assessed on these axial planes per a modified classification system of Frodel et al. [16] (Fig. 1C). Finally, the aspect ratio between the anterolateral (Fig. 1C: 7, 9) and the postero-lateral (Fig. 1C: 8, 9) surface of the fibula was calculated to assess the symmetry of the distal fibula.

### Fibula safe zones for drilling-tunnel

**Fig. 2:**
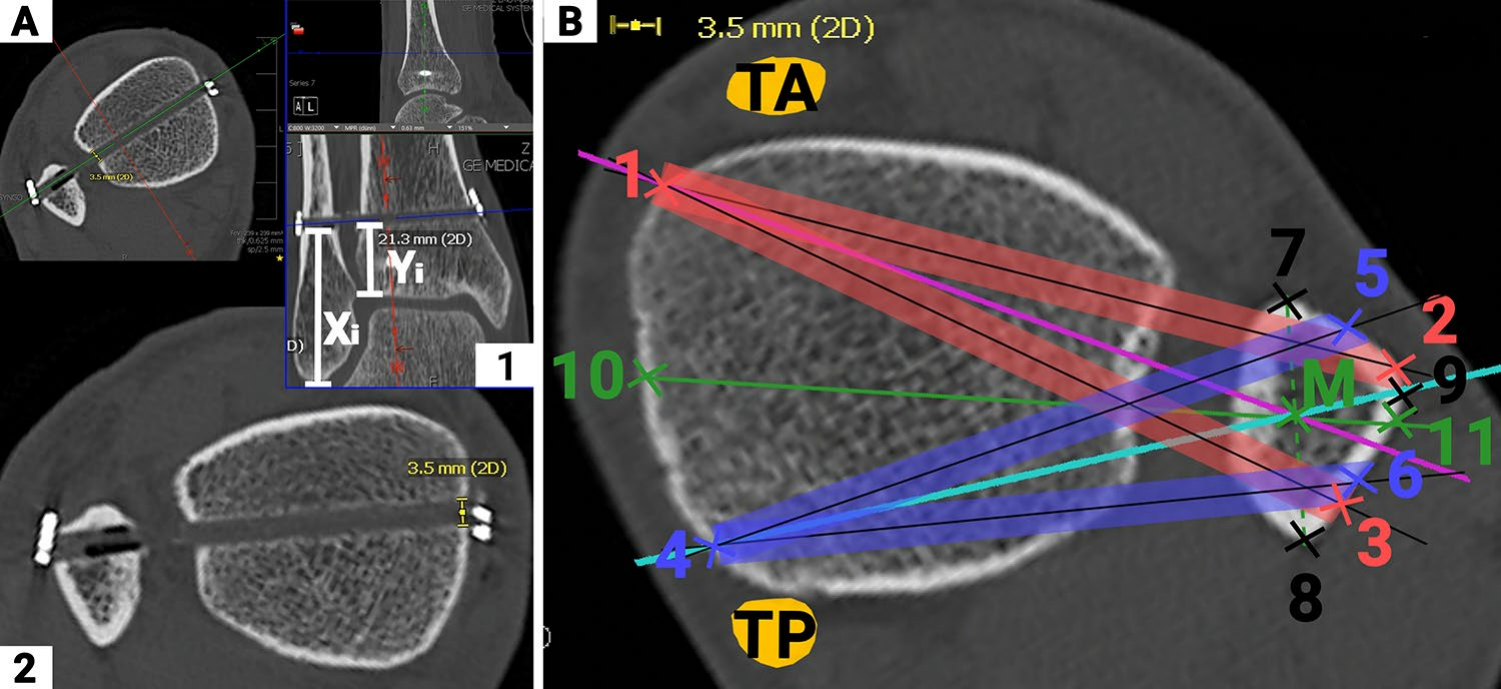
Illustration of the measurements conducted to define the fibula safe-zones for the different drilling-tunnel orientations. **A** CT reconstructions of injured side with the central alignment of the suture-button drilling tunnel and width/height measurements, Yi: height of the measurement location from the distal lateral pilon tibial suture-button location; Xc: height of the measurement location from the distal tip of the fibula to the suture-button location. **B** Contralateral CT reconstruction illustrating the measurement conducted on the axial slices: 1: medial anterior landing zone for anteriorly angulated drilling tunnel, just medial to the anterior tibial tendon 2: boarder of the most anteriorly angulated anterior drilling tunnel, 3: boarder of the most posteriorly angulated anterior drilling-tunnel, red rectangle: anterior drilling-tunnels; 4: medial posterior landing zone for the posteriorly angulated drilling-tunnel, just anterior to the posterior tibial tendon grove; 5: boarder of the most anteriorly angulated posterior drilling-tunnel, 6: boarder of the most posteriorly angulated posterior drilling-tunnel, blue rectangle: posterior drilling-tunnels; 7: anterior cortical apex; 8: posterior cortical apex; 9: lateral cortical apex

A more detailed description of the methodology applied is given in Supplement 1. First, the maximum drilling tunnel diameter was measured (Fig. 2A) and its location (height) assessed as outlined above (Fig. 2A1: Yi, Xi). The drilling-tunnel simulations were based on these measurements and conducted in Adobe Photoshop (Vs. 23.2.2, Dublin, Republic of Ireland) for each ankle separately. Three drilling-tunnel orientations were simulated, i.e. anteriorly angulated (Fig. 2B: lines 1–2 and 1–3), posteriorly angulated (Fig. 2B: lines 4–5 and 4–6), and center-center (Fig. 2B: lines 10–11). Each drilling-tunnel simulation comprised a defined medial anchor point (landing zone) and individual locations on the lateral fibula. The anteriorly and posteriorly angulated drilling-tunnel orientations were based on the medial anchor points (anteriomedial: Fig. 2B: point 1; posteromedial: Fig. 2B: point 4) and the most anterior (Fig. 2B: lines 1–2 and 4–5) and posterior (Fig. 2B: Lines 1–3, 4–6) drilling tunnel locations possible. Marked were the intersections of the lateral fibula cortex and the longitudinal axis (K-wire) of the most anterior (Fig. 2B: points 2 and 5) and posterior drilling tunnel locations (Fig. 2B: points 3 and 6). Second, the ideal anterior and posterior drilling-tunnel positions were simulated through the bisector of the fibular width (point M). Finally, the center–center drilling-tunnel orientation was simulated as a straight line defined through the bisectors of the anterior and posterior tibial landing zone (Fig. 2B: Point 10) and most anterior (Fig. 2B: point 7) and posterior (Fig. 2B: point 8) apex of the fibula.

Then, these images were imported into ImageJ (Vs. 13.0.6, National Institutes of Health, USA). In ImageJ, the previously defined points (Fig. 2B: points 1–6, 10, 11) and the most anterior, posterior and lateral apex of the fibula (Fig. 2B: points 7–9) were marked and the individual x-y coordinates exported to excel.

**Fig. 3:**
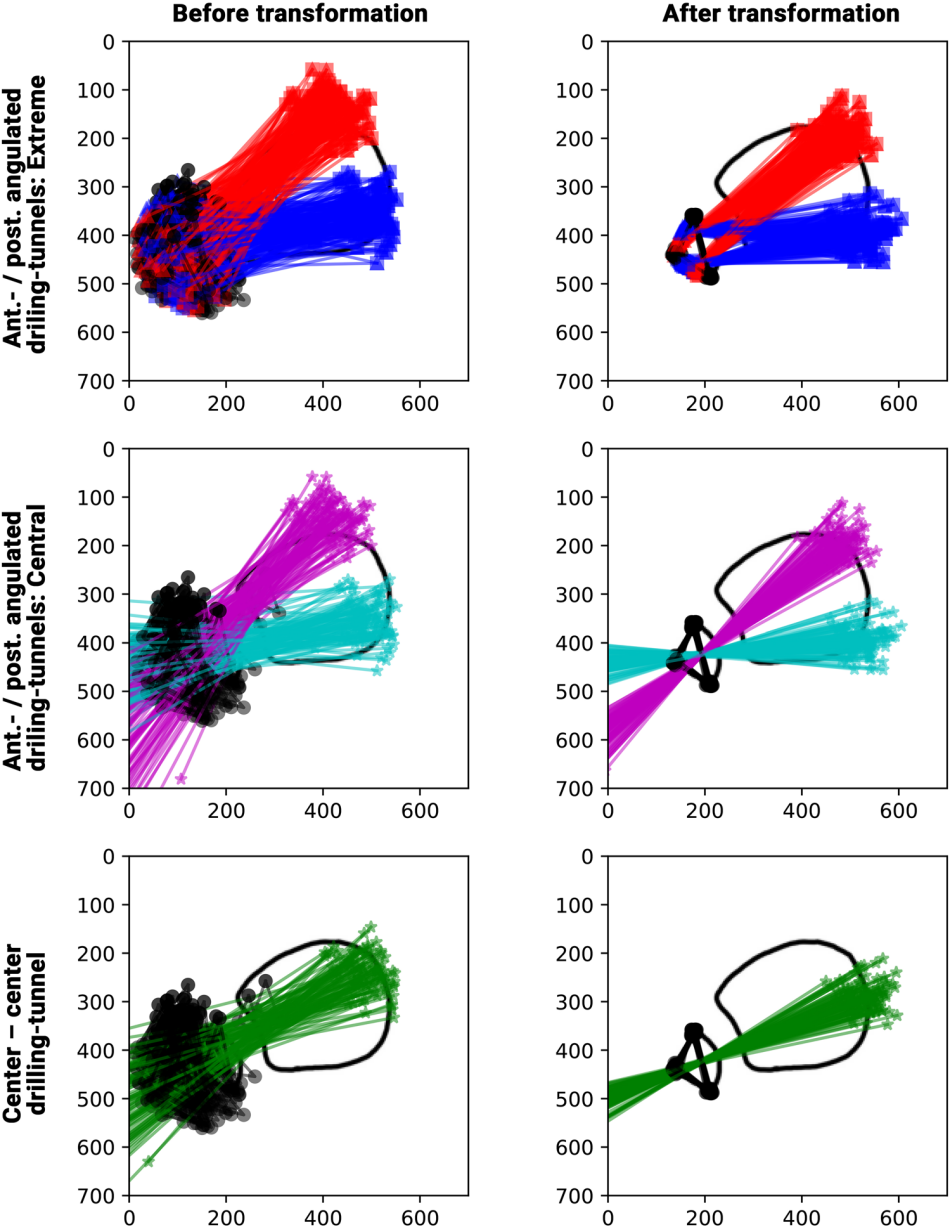
Illustration of the data analysis and registration process. All annotated points and simulated drilling tunnels are shown before (left) and after (right) registration and transformation to the reference image based on the fibular points (black points and triangles). The outline of the tibia and fibula of the reference image is indicated as a black line in the background. *Ant.* Anteriorly, *post*. posteriorly, *extreme* anterior and posterior boards of the drilling tunnels, *central* central orientation of the anteriorly (pink) and posteriorly (light blue) angulated drilling tunnels, *red lines* anteriorly angulated drilling tunnels*, dark blue lines* posteriorly angulated drilling tunnels, *green lines* center–center drilling-tunnels

A custom python script was used to transform all coordinates to a reference image based on the fibula coordinates (Fig. 2B: Points 7–9, Fig. 3) using Procrustes analysis [17].

### Data analysis

First, the drilling-tunnel heights were compared between the operated and simulated sides. Then, the different anatomical shapes of the fibula were analyzed as well as the aspect ratio of the anterio-lateral (Fig. 2B: 7, 9) and the posterio-lateral (Fig. 2B: 9, 8) fibula surfaces. Finally, the images were analyzed per the quality of registration using the mean distance of the registered fibula points with respect to the reference fibula.

**Fig. 4:**
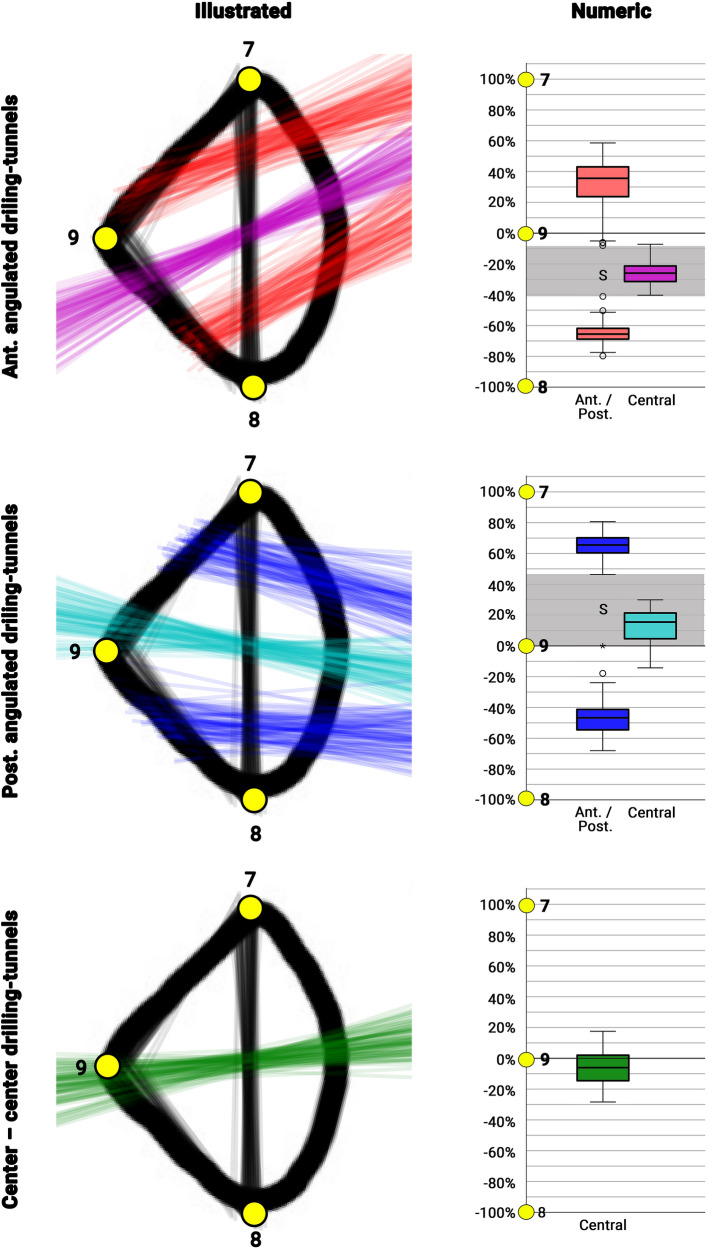
Illustration and numeric representation of the anteriorly and posteriorly angulated drilling tunnels and the center-center drilling-tunnel orientations on the anterior and posterior surface of the lateral fibula. Numeric: drilling-tunnel locations illustrated as percentage of the anterior (Points 7, 9) and posterior (Point 9, 8) surface of the lateral fibula; red: anterior and posterior borders of the anteriorly angulated drilling-tunnels; pink: central orientation of the anteriorly angulated drilling-tunnels; dark blue: anterior and posterior borders of the posteriorly angulated drilling-tunnels; Light blue: central orientation of the posteriorly angulated drilling-tunnels; green: center–center drilling-tunnel orientation; 7: anterior apex of the fibula; 8: posterior apex of the fibula; 9: lateral apex of the fibula

Second, a visual representation of the most extreme anterior-, posterior borders, the ideal orientation of the anteriorly and posteriorly angulated drilling tunnels as well as the center-center drilling tunnels on the lateral fibula were generated. Third, this visualization was quantified by calculating the location of the intersection points of the anterior (Fig. 2B2: 7, 9)/posterior (Fig. 2B2: 8, 9) fibula cortex and the individual drilling tunnels’, using custom Python scripts. The location was normalized based on the length of the respective cortical edge, and locations on the anterior and posterior cortex were assigned positive and negative values, respectively. Finally, a safe corridor was calculated for each drilling-tunnel orientation. A safe corridor was defined as the area at the lateral fibula cortices, which could safely be used as a drilling starting point without perforating the anterior or posterior fibula cortex. This was done separately for the anterior, central, and posterior drilling tunnel (Fig. 4). The anterior and posterior safe corridor was defined by the minimum of the anterior- and the maximum of the posterior drilling-tunnel border. The central safe corridor was defined by the most anterior and posterior drilling tunnel.

### Statistical analysis

The primary analysis performed was descriptive. Values are given as mean ± standard deviation (range), if not stated differently. Further comparisons were performed using the paired t-test and Pearson-Correlation, where appropriate. Due to the retrospective nature of the study, no posthoc power analysis was conducted. All statistical analyses were performed using IBM^®^ Statistical Package for the Social Sciences (SPSS^®^), version 28.

## Results

### Shape of the distal fibula

The height of the simulated drilling tunnels from the tibial plafond (Fig. 1B3: Yc)/tip of the distal fibula (Fig. 1B3: Xc) on the uninjured side were 19 ± 3 mm (range: 14–25 mm)/44 ± 4 mm (range: 34–53 mm). The vast majority (81%) of fibulae revealed a triangular convex shape, 10% an irregular shape, and 8% a quadrilateral shape at the desired level of the suture-button system. Consequently, 89% of the axial cross-sections showed a configuration with a clear anterior, lateral and posterior apex. The ratio between the anterio-lateral and posterior-lateral fibula surface was 1.0 ± 0.2 (range: 0.7–1.5). The ratio did not differ between the different fibula types (n.s.).

### Fibula safe zones for drilling-tunnel

The height of the simulated drilling tunnels from the tibial plafond (Fig. 2A1: Yi)/tip of the distal fibula (Fig. 2A1: Xi) was significantly more distal compared to the actual drilling-tunnel height on the operated sides (23 ± 6 mm (range: 7–48 mm)/48 ± 7 mm (range: 32–72 mm); *p* < 0.001). Correlating the patient height to the height of the drilling tunnel, showed a significant moderate correlation for the simulated, i.e. contralateral, side (*r* = 0.421; *p* < 0.001/*r* = 0.415; *p* < 0.001) but no correlation for the height on the operated side (*r* = 0.132; n.s./*r* = 0.220; n.s.).

All images could be registered with a mean distance error of 0.5 ± 0.2 mm (range: 0.1–1.2 mm). Figure 3 depicts the results for the anteriorly and posteriorly angulated drilling tunnels and the center-center drilling-tunnel orientations, before and after transformation. The quantitative analysis is shown in Fig. 4. For an anteriorly angulated drilling tunnel the safe corridor (Fig. 4: S) was between − 8% and − 41% of the lateral surface of the fibula, and the average central alignment was − 26 ± 7% (range: − 40 to − 7%). For a posteriorly angulated drilling tunnel the safe corridor (Fig. 4: S) was between 0 and 46% of the lateral surface of the fibula, and the average central alignment was 13 ± 10% (range: − 14 to 30%). For a center-center alignment, the average location on the lateral surface of the fibula was − 7 ± 11% (range: − 28 to 18%).

## Discussion

The most important findings of the present study were a uniform, triangular-like cross-sectional shape of the distal fibula with a lateral aspect ratio of almost 1:1 and the definition of fibular safe-corridors for an anterior, central, posterior drilling tunnel. The lateral apex of the fibula can thereby serve as a reliable landmark.

### Measurement location

Previous studies have recommended placing the syndesmotic stabilization device 20–30 mm proximal and parallel to the tibial plafond [5–8]. Placement above 40 mm has been shown to result in inferior outcomes [18]. The actual drilling-tunnel location on the operated side was well within this range (23 ± 6 mm) but with a considerable variation (7–48 mm). Since 56% of the patients included suffered an ankle fracture combined with syndesmotic injuries this variation might be explained by the predefined spacing of the plate holes and fracture characteristics of the fibula. The simulated heights of the drilling tunnels, at the proximal end of the articulation between the distal tibia and fibula, defined on individual CT reconstructions, were significantly lower (19 ± 3 mm (range: 14–25 mm)) but in line with previous in-vivo studies [19]. Biomechanical studies demonstrated a higher primary stability for syndesmotic screws placed at 20 mm compared to 30 mm above the tibial plafond [20]. Moreover, a significant correlation between the patients’ height and the simulated but not the actual drilling-tunnel location could be observed. This linear bone geometry dependency has been demonstrated for other anatomical locations [21]. Consequently, surgeons should rather respect the individual anatomy and should be guided by anatomical landmarks, instead of apodictically following predefined values [19].

### Shape of the distal fibula

The prerequisite of any further analysis and generalization of the findings on the whole sample was a uniform shape of the distal fibula. Previous studies had analyzed the cross-sectional shape of diaphyseal fibulae at various locations (Fig. 1A: C–E). But no study had investigated the shape of the fibula at the proximal end of the articulation between the distal tibia and fibula, the location where a syndesmotic stabilization device is implanted (Fig. 1A: F). While the shape of the diaphysis of the fibula has a considerable, gender dependent, heterogeneity [11–14], in the meta-diaphyseal region analyzed a very uniform configuration of the fibula was observed. The vast majority of fibula had a triangular convex shape, i.e. a lateral-based isosceles triangle (aspect ratio: 1.0 ± 0.2) with a convex hypotenuse. This convexity, other than the predominantly triangular, quadrilateral, or irregular shape of the diaphyseal fibula, appears reasonable, as it forms the counterpart to the concave incisura of the tibia. Due to the uniformity of the shape of the meta-diaphyseal fibula, the pooled drilling tunnel analyzes could be performed. The mean registration accuracy of 0.5 ± 0.2 mm (Range: 0.1–1.2 mm) further proofs the uniform presentation of the defined landmarks.

### Fibula safe zones for drilling

Various authors have tried to define strategies to guide syndesmotic screw placement aligned along the central syndesmotic axis, i.e. centrally through the fibula and tibia, using CT [7, 22] or MRI image analysis [23]. All studies identified anatomical landmarks and defined the subsequent drilling-tunnel angulation. The suggested landmarks varied from positioning the second toe vertically [7] to imagining a line through the Anterior Tibial and Achilles tendons [22] with subsequent angulations of 18.8° ± 5.6° [7] and 26.2° ± 1.1° [22]. A more applicable recommendation was published by Kumar et al. [23]. They analyzed 70 CTs of uninjured normal ankle joints and found that the malleolar tips are reliable anatomical landmarks to guide syndesmotic screw placement with an observed deviation of 3.70 ± 5.61°. No study has yet suggested guidelines for an angulated syndesmotic stabilization, i.e. individually supporting the injured syndesmotic aspects.

In the present studies, a different approach was facilitated. The idea was to put the cart before the horse. Therefore, the first easily identifiable tibial landing zones were defined. From there subsequently safe zones within the anterio lateral and the posterior-lateral surface of the distal fibula were identified. Due to the triangular shape of the fibula at the level of syndesmotic stabilization, the lateral cortical apex of the fibula can serve as an easy applicable landmark to allow for orientation. Placing the guide K-wire within these safe zones and aiming at the subsequent tibial landing zone ensures, that the drilling tunnel will not weaken the anterior or posterior cortex of the fibula. The tibial landing zones were chosen, to be easily identifiable and to avoid anatomical structures at risk. The anterior tibial landing zone was located just medial to the anterior tibial tendon. Studies have indicated that the greater saphenous neurovascular bundle is located more than 10 mm medial to the anterior tibial tendon [24]. The posterior tibial landing zone was defined just anterior to the posterior tibial tendon grove. The posterior tibial neurovascular bundle is posterior to the posterior tibial tendon and therefore not at risk [25]. Solely the central placement bears the risk of entrapment of or damage to the greater saphenous neurovascular bundle. Therefore, an additional medial incision should be carried out to ensure safe positioning [26, 27].

Several limitations should be discussed. First, the analysis was based on bilateral CT images of unilateral ankle fracture cases treated surgically, not on healthy individuals. Still, all CT scans of the uninjured side were assessed for any posttraumatic changes or anatomical variations, which were subsequently excluded from further analysis. This had the advantage, that the individual drilling-tunnel diameter could be assessed on the injured side and therefore account for scaling effects. Second, the cross-sectional morphology of the fibulae was not uniform, with 10% presenting an irregular and 8% a quadrilateral shape. Still, the predominant landmarks, i.e. the anterior-, lateral-, and posterior cortical apexes could be identified in all cases. Furthermore, the registration algorithm resulted in mean accuracy of 0.5 ± 0.2 mm (range: 0.1–1.2 mm), resembling a good fit for all specimens. Finally, the analysis was performed for a 3.5 mm drilling tunnel. Consequently, the identified safe zones do not apply, if a syndesmotic stabilization devices is used with a greater diameter.

Despite these limitations, the large study cohort, the analysis of three different drilling-tunnel orientations, i.e. anteriorly, posteriorly angulated and center-center, and the automated registration of the images and analysis of the drilling tunnels are considerable strengths of the current study.

## Conclusion

The meta-diaphyseal region of the distal fibula revealed a considerable homogeneous cross-sectional shape. The lateral apex of the fibula can serve as an easily applicable landmark for the intraoperative placement of the drilling tunnels oriented either anteriorly, posteriorly or center-center. Using this reference point, safe zones could be defined to safely place the drilling tunnels without weakening the cortex of the fibula. The K-wire for an anteriorly oriented suture-button-system should be placed just posterior, for a posteriorly oriented suture-button-system just anterior to the lateral apex of the fibula. In case a center-center alignment is desired, the K-wire should be placed at, or just posterior to the lateral apex. Applying these safe zones in clinical practice could help to avoid the misplacement of the syndesmotic fixation device.

## Supplementary Information

Below is the link to the electronic supplementary material.

Supplementary file1 (DOCX 16 KB)

## Data Availability

The data is available on request.
