# Supplementary material for: Safe drilling zones for anteriorly, central, and posteriorly angulated syndesmotic stabilization devices
Source: Knee Surg Sports Traumatol Arthrosc. 2022 Dec 22;31(6):2199–207. doi: 10.1007/s00167-022-07291-x (PMC10183421; doi:10.1007/s00167-022-07291-x)
Supplement: Supplementary file 1 — Supplementary file1 (DOCX 16 KB) [file 167_2022_7291_MOESM1_ESM.docx]

(1) Shape of the distal fibula

The shape of the distal fibula was evaluated on the postoperative CT images of the contralateral, uninjured side. At the level of the ankle joint, all CT data sets were manually reconstructed in all three planes, with the coronal plane positioned in the malleolar axis (Fig. 1B1), the sagittal plane aligned with the axis of the tibia (Fig. 1B2), and the axial plane parallel to the joint line (Fig. 1B3). Then, an axial slice parallel to the tibial plafond was generated at the preferred location for a suture-button system, namely just proximal to the articulation between the distal tibia and fibula (Fig. 1B). The location (height) of the defined axial slices was then measured from the most lateral aspect of the tibia plafond in the coronal plane (Fig. 1B3: Yc) and from the tip of the distal fibula (Fig. 1B3: Xc). All reconstructed images were saved as .jpg files.

Next, the shape of the distal fibula was assessed on these axial planes. Classification was conducted in a modification to the proposed classification system of Frodel et al. [4], as outlined in Figure 1C. Furthermore, the aspect ratio between the antero-lateral (Fig. 1C: 7,9) and the postero-lateral (Fig. 1C: 8,9) surface of the fibula was calculated to assess the symmetry of the distal fibula. To do so, the most anterior (Fig. 1C: 7), posterior (Fig. 1C: 8) and lateral (Fig. 1C: 9) apex of the fibula were marked. All measurements were conducted by FTS and reviewed by a senior foot and ankle surgeon (SFB). In case of disagreement, the measurement location was adapted following agreement between FTS and SFB.

(2) Fibula safe-zones for drilling-tunnel

First, the postoperative CT images of the operated sides were manually reconstructed so that the suture-button drilling-tunnel was fully visible at its maximum diameter in all three planes (Fig. 2A). Then, the maximum drilling-tunnel diameter was measured (Fig. 2A2) and its location (height) was assessed as outline above (Fig. 2A1: Yi, Xi).

The previously saved axial slices of the uninjured side were employed to define the fibula safe-zones (Fig. 2B). The drilling-tunnel diameter measurement of the operated side had been transferred to these images to avoid possible scaling effects. The axial slices were exported to Adobe Photoshop (Vs. 23.2.2, Dublin, Republic of Ireland). Three drilling-tunnel orientations were simulated, i.e. anteriorly angulated (lines 1-2 and 1-3), posteriorly angulated (lines 4-5 and 4-6), and center-center (lines 10-11). Each drilling-tunnel orientation comprised of a defined medial anchor point (landing zone) and individual locations on the lateral fibula.

The anteriorly- and posteriorly angulated drilling-tunnel orientations were simulated first. The anterior tibial landing zone was located on the anteromedial curvature of the tibia, just medial to the anterior tibial tendon (point 1). The posterior tibial landing zone was located on the posteromedial curvature of the tibia, just anterior to the posterior tibial tendon grove (point 4). Each simulated drilling-tunnel was resembled by a rectangle with the individually measured, contralateral drilling-tunnel diameter and a central line simulating the guide K-wire. First, the most extreme anterior and posterior drilling-tunnel positions were assessed by anchoring the individual drilling-tunnel at the medial landing zones and then rotating it into the fibula so that the anterior (point 7; Lines 1-2 and 4-5) or posterior cortex (point 8; Lines 1-3 and 4-6) were just not intercepted. Second, the ideal anterior and posterior drilling-tunnel positions were simulated. Therefore, the same tibial anchor points were used but the drilling-tunnel was placed through the bisector (point M) of the most anterior (Point 7) and posterior (Point 8) fibular apex, i.e. central through the fibula.

Then the center–center drilling-tunnel orientation was simulated. This time, only the guide K-wire was simulated by a straight line defined by the following two points: Bisectors between the anterior and posterior tibial landing zone (Point 10) and most anterior (Point 7) and posterior (Point 8) apex of the fibula.

Thereby each drilling- tunnel was clearly defined by a medial (medial tibial landing zone; Points 1, 4, 10) and a lateral (intersection K-wire and lateral cortex of the fibula; Points 2, 3, 5, 6, 11) point and represented by a line (resembling the guide K-wire). These lines were used for any further analysis. This simulation was performed for each individual patient on the axial CT slice and again saved as .jpeg files.

Then, these images were imported into ImageJ (Vs. 13.0.6, National Institutes of Health, UAS). In ImageJ, the previously marked points (Fig. 2B2: Points 1-6, 10, 11) and the most anterior, posterior and lateral apex of the fibula (Fig. 2B2: Points 7-9) were marked and the individual x-y coordinates exported to excel.

A custom python script was used to transform all coordinates to a reference image based on the fibula coordinates (Fig. 2B2: Points 7-9, Fig. 3). First, an arbitrary image was selected as reference. Then, a Procrustes analysis [5] was performed to find the rotation, translation and scaling that minimized the squared distance between the fibula coordinates of the reference image and a given patient image. Then, all points (Fig. 2B2: Points 1-11) were transformed by applying the optimal rotation, translation and scaling. The process was repeated for each patient image.
